# Technology and Data Implications for the Public Health Workforce

**DOI:** 10.1089/big.2022.0208

**Published:** 2022-09-06

**Authors:** Laurie T. Martin, Anita Chandra, Christopher Nelson, Douglas Yeung, Joie D. Acosta, Nabeel Qureshi, Tara Blagg

**Affiliations:** ^1^ RAND Corporation, Arlington, Virginia, USA.; ^2^ RAND Corporation, Santa Monica, California, USA.; ^3^ Pardee RAND Graduate School, Santa Monica, California, USA.

**Keywords:** public health, workforce, equity

## Abstract

Achieving a modern equity-oriented public health system requires the development of a public health workforce with the skills and competencies needed to generate findings and integrate knowledge using diverse data. Yet current workforce capabilities and infrastructure are misaligned with what is needed to harness both new and older forms of data and to translate them into information that is equity contextualized. As with other articles in this supplement, this article builds from a literature review, environmental scan, and deliberations from the National Commission to Transform Public Health Data Systems. The article summarizes some of the challenges around current workforce capabilities and pipeline. The article identifies where the technology and data sectors can contribute skills, expertise, and assets in support of innovative workforce models and augment the development of public health workforce competencies.

## Introduction

Critical to the success of a modern equity-oriented public health system is a public health workforce with the skills and competencies needed to generate findings and integrate knowledge using diverse data contextualized through an equity lens. Yet, the skills and abilities of the current, formal public health workforce have not kept pace with rapidly emerging technologies, a growing understanding of the multifaceted and multigenerational impacts of inequity on health, and new ways of working that leverage community voice and power.

Under-resourced departments^1^ have contributed to this widening gap, as—even before the COVID-19 pandemic—public health professionals left the field for private industry and better-resourced positions. The state public health workforce decreased by an estimated 10% between 2012 and 2018,^2^ with an even greater reduction in the public information workforce (−33%) and public health informatics workforce (−29%) between 2010 and 2013.^3^ Collectively, this has resulted in significant variability in public health workforce capacities and competencies, which are even more variable at the local level.^4,5^

In this article, we examine whether the public health workforce has the skills, competencies, and expertise to effectively leverage an equity-oriented data system to develop localized innovative solutions for emerging health concerns. We also discuss the current workforce pipeline and question whether current education and training go far enough on critical topics, including decision-making under deep uncertainty, community engagement, and organizational partnership. To address more immediate needs, this article also elevates the potential for innovative models through public–private partnerships or cooperative agreements that may supplement and enhance current workforce capabilities. We conclude with a discussion of the skills, expertise, and assets that the data science and technology sectors could bring to this effort in support of innovative workforce models and augmented development of public health workforce competencies.

## Methods

In 2020, the Robert Wood Johnson Foundation (RWJF) formed the National Commission to Transform Public Health Data Systems to review significant challenges to the current public health data system, and “provide recommendations to policymakers, health care organizations and institutions, service providers, and philanthropy” on potential solutions to overcome these challenges.^6^

In support of this effort, RAND conducted an environmental scan to identify key issues, points of consideration, tradeoffs and tensions, and current activities related to public health data, data systems, and data modernization efforts. This effort included a targeted scan of published research articles and reports, reviews of websites and working documents describing coordinated activities (e.g., data interoperability), and recent initiatives. Additional searches included the use of “big data” in public health, data privacy, and ethics of public health data collection. Although the team focused on public health data, it also identified seminal articles and reports from other sectors or disciplines whose findings could apply to public health data systems.

RAND simultaneously conducted semistructured interviews with 112 experts and thought leaders on the main topics before the Commission. Individuals represented diverse sectors, including public health and health care, technology and data science, research and policy, journalism, and law. The interviews also included experts in data, data use, equity, community engagement, and research translation who work *outside* the traditional health sector. The project was reviewed and approved by the RAND Human Subjects Protection Committee.

In this article, we highlight relevant findings from this supporting analysis and then implications for the data science and technology sectors, with consideration of recommendations that emerged from the final Commission report.

## Findings

Current workforce capabilities and infrastructure are misaligned with what is needed to harness both new and older forms of data and to translate them into information that is equity contextualized. Several recommendations and action items emerged from Commission deliberations related to strengthening the public health workforce. These include increasing data literacy and strengthening competencies in informatics and analysis of complex data.^6^ Commission deliberations also elevated a gap—both within and outside the public health workforce—around the need for a deeper understanding of equity principles and a willingness to tackle more complex health challenges that are shaped by historical policies and practices.^6^

Given challenges in recruiting and maintaining top talent within the public health workforce, the Commission discussed whether there is an opportunity for a narrative shift that highlights important challenges and unique opportunities within the field, enticing those looking to become trailblazers in these emerging areas. Workforce exchanges and cross-sector models of job-sharing outside of traditional public health also were offered as a more near-term solution that could supplement public health competencies and fill these gaps.

### The current public health workforce may not have the skills and competencies necessary to operate in a modern information age

There is growing concern that the public health workforce, in general, may not have the skills and competencies necessary to efficiently operate in a modern information age. Although some public health agencies, particularly those at the state or well-resourced city or county levels, have a workforce with more diverse expertise, including those that are highly skilled at data manipulation and informatics, such expertise is often not available at many local levels.^7^

Individuals skilled in informatics are choosing private industry over public health, given the opportunity for better pay and advancement. In some cases, there is no job classification specific to informatics, making it difficult for public health departments to attract the right candidates.^7^ Observed gaps in skills and competencies within and across public health have prompted inclusion of workforce education and training as an important action item in both the Centers for Disease Control's Data Modernization Initiative^8^ and the Council for State and Territorial Epidemiologists 2019 report.^9^

These concerns also raise a larger consideration around whether there should be formalized competencies for public health data stewards more broadly, and whether public health professionals are equipped for this role. Data stewards of the future, for example, will need to have not only data governance expertise but also expertise in data management and informatics.^6,7^ There is an opportunity now to consider in more detail what competencies the data stewards of the future will need, how to ensure we collectively develop the data stewards of the future through education and training programs, and whether there is an opportunity to standardize the position through a certification program or equivalent.^6,7^

### The future public health workforce must understand equity and the multifaceted and multigenerational impacts of inequity on health

A transformed public health *data* system must reflect a modern public health system, one that recognizes the health needs of the future. Achieving a modern equity-oriented data system requires widespread acknowledgement of factors driving health and well-being in the community today, including, for example, information on historical systems and structures that have resulted in variable opportunities for health, and not merely disease mitigation and cost containment.^7^ A critical question, however, is whether the public health workforce has the skills and capacity to leverage a more robust equity-centered data system. For example, the COVID-19 pandemic highlighted a need for public health workers to have expertise in decision-making under deep uncertainty^*,10^ as well in using diverse and potentially messy data sources for sense-making, some of which may be incomplete or evolving during public health emergencies.^11–13^

There is also a need to train the next generation of public health practitioners and researchers to meaningfully partner with local stakeholders, and to value lived experience and community expertise as much as formal training in the design, implementation, collection, interpretation, and dissemination of data and public health solutions.^7,14^ At the same time, faculty and researchers have an opportunity to assess their own biases and approaches to working and consider ways in which their work and their interactions with students could contribute to a modern equity-oriented public health data system.^14^ This opportunity is not limited to schools of public health but includes allied fields such as schools of public policy.

Although efforts such as the Association of Schools and Programs of Public Health *Framing the Future*^15^ have focused on new capacities needed for the field, it is unclear how well new data skills—from equity orientation to how to translate data into information and insight—have been imbued into the workforce.^14^ For instance, is the current workforce pipeline producing graduates who understand equity and its multifaceted and multigenerational impacts on health? Are graduates taught to then apply that understanding to meaningfully engage with community stakeholders on sense-making and decision-making activities to ensure diverse perspectives are not lost?

Does student training include how to balance the precision of research with more rapid sense-making using data that may be necessary in a public health emergency and for which data may be less precise? Does student instruction place value on the diversity of public health data, including qualitative data and authentic lived experiences of those within the community in which they will be working? Collectively, these questions point to a diverse set of substantive and methodological skills and expertise that future public health practitioners and leaders will need to possess.

### Workforce exchanges and cross-sector models of job sharing could supplement and strengthen capacities of current public health practitioners while providing reciprocal benefits

Public health faces a perfect storm of (1) a public health workforce whose skills and competencies fall short of what will be needed to leverage an equity-centered public health data system, (2) rapidly evolving technology and data systems that place a heavier emphasis on data analytics, and (3) a resource-constrained environment that precludes meaningful infrastructure or personnel investments. Although formal education and pipeline programs are working to train the next generation of public health practitioners, variability across these programs means that the competencies of future public health practitioners will continue to vary.

A shorter-term solution to filling public health gaps in both capacity and skill may be to leverage innovative models of cross-sector collaboration that would allow public health practitioners to tap into the expertise of certain individuals on a regular basis. For example, more consistent workforce exchanges with public health organizations might cross-pollinate innovations in health data content and application.^7^ Establishing such exchanges with local organizations, companies, or schools of higher education may have a secondary benefit of increasing the leadership of underrepresented communities and populations. Another model may be to provide more centralized or regional support for more complex analytic needs, rather than to require such expertise locally, which could alleviate challenges related to availability of resources and local talent.^7^

## Implications

The workforce considerations discussed earlier revolve around two main challenges: competencies needed to leverage an equity-centered public health data system efficiently and effectively, and the capacity to do so. Data science and technology companies are well poised to help address both challenges. One of the Commission's calls to action noted the opportunity for businesses to support the transformation of our public health data system through workforce exchanges and partnerships to “cross-pollinate innovations in the types, content, quality, and precision of public health data.”^6^ We highlight two major implications for this sector here.

### Data science and technology companies could inform development of a competencies framework for the public health workforce and other users of the data system

As noted, there is growing concern that the public health workforce lacks the core skills to optimize information technology. With this comes a need to develop a set of core competencies or a competencies framework to increase data literacy and ensure that public health practitioners and leaders have both an understanding of and ability to work with diverse types of data.^6,14^ Data science and technology companies have a unique lens into the types of competencies and skills that may be needed, as well as insight on the types of data analytics innovations that may be relevant in the future. The data science and technology sector could help to parse out what types of skills should become core competencies and why and offer strategies and solutions to build those core competencies for the existing and future public health workforce.

### Data science and technology companies could engage in workforce exchanges and data partnerships with public health organizations

New models of public–private partnerships or cooperative agreements with data science and technology companies could augment the quality and precision of data content and use. Such companies have a wealth of talent in informatics, “big data” analytics, and data security, and are often at the cutting edge of new technologies and approaches to finding signal value within vast amounts of data.^6,14^ Although the structure of such partnerships or agreements is likely to vary, depending on the needs and capabilities of the public health department, such arrangements have an added benefit of strengthening relationships between sectors and could prove mutually beneficial.

For example, not only could such arrangements expand a company's reach and network, but public health colleagues may also provide insight into specific challenges that require more intensive methodological or product innovation. This real-world insight into such challenges could inform future directions within a technology company, resulting in a win-win for both parties.

## Conclusion

For the past 150 years, the field of public health has experienced several “waves” of focus, starting with an understanding of the link between unsanitary conditions and health.^16^ This understanding of the causes of health expanded in the second wave, with the introduction of hospitals, medical experts, and theories of disease that remain today. In wave three, we saw a greater appreciation for how our lifestyle and everyday lives impact our health, and in wave four more recognition was given to policies and systems that shape health and well-being. In 2011, the concept of a fifth wave was introduced^16^ in response to alarming trends in social inequalities, and rises in mental health concerns, among others, that point to the need for a multisector approach to building a “health-promoting social context.”^17^

Despite an evolving understanding of the factors that interact to produce health, public health workforce skills and capabilities have not kept pace with this growth in knowledge. Input from the literature and experts, coupled with Commission deliberations, elevate this gap as critically important for the success of an equity-oriented public health data system. The data science and technology sector is positioned to help elucidate a new set of core competencies and increase capacities to help alleviate this gap both now and for the long term.

## Abbreviation Used

RWJF: Robert Wood Johnson Foundation

## Notes

^*^
Deep uncertainty refers to the level of uncertainty of the event itself or to the level of uncertainty we have about our own knowledge. Deep uncertainty relates to a situation in which events (e.g., catastrophic) cannot be addressed by assigning probabilities to its occurrence.

## References

[1] Maani N, Galea S. COVID-19 and underinvestment in the public health infrastructure of the United States. Milbank Q 2020;98(2):250; doi: 10.1111/1468-0009.12463. 32333418 PMC7296430

[2] Carlin M, Ensign K, Person CJ, et al. State of the public health workforce: Trends and challenges leading up to the COVID-19 pandemic. J Public Health Manage Pract 2021;27(1):92–93; doi: 10.1097/PHH.0000000000001294. 33239529

[3] Beck AJ, Boulton ML. Trends and characteristics of the state and local public health workforce, 2010–2013. Am J Public Health 2015;105(S2):S303–S310; doi: 10.2105/AJPH.2014.302353. 25689210 PMC4355719

[4] Trust for America's Health. The Impact of Chronic Underfunding on America's Public Health System: Trends, Risks, and Recommendations. Washington, DC; 2020. Available from: https://www.tfah.org/report-details/publichealthfunding2020 [Last accessed: March, 2022].

[5] DeSalvo K, Parekh A, Hoagland GW, et al. Developing a financing system to support public health infrastructure. Am J Public Health 2019;109(10):1358–1361; doi: 10.2105/AJPH.2019.305214. 31415208 PMC6727291

[6] National Commission to Transform Public Health Data Systems. Charting a Course for an Equity-Centered Data System: Recommendations from the National Commission to Transform Public Health Data Systems. Robert Wood Johnson Foundation: Princeton, NJ; 2021. Available from: https://www.rwjf.org/en/library/research/2021/10/charting-a-course-for-an-equity-centered-data-system.html [Last accessed: April, 2022].

[7] Martin L, Chandra A, Acosta J, et al. Transforming Public Health Data Systems, How? The Design of the Modern Public Health Data System. Robert Wood Johnson Foundation, National Commission to Transform Public Health Data Systems: Princeton, NJ; 2021.

[8] Centers for Disease Control and Prevention. Data Modernization Initiative: An Urgent Need to Modernize. Atlanta, GA; 2020. Available from: https://www.cdc.gov/surveillance/surveillance-data-strategies/dmi-investments.html [Last accessed: April, 2022].

[9] Council of State and Territorial Epidemiologists. Driving Public Health in the Fastlane: The Urgent Need for a 21^st^ Century Data Superhighway. Atlanta, GA; 2019. Available from: https://cdn.ymaws.com/www.cste.org/resource/resmgr/pdfs/pdfs2/Driving_PH_Print.pdf [Last accessed: March, 2022]

[10] Lempert RJ, Popper SW, Bankes SC. Shaping the Next One Hundred Years: New Methods for Quantitative, Long-Term Policy Analysis. RAND Corporation: Santa Monica, CA; 2003. Report No.: MR-1626-RPC.

[11] Barbero C, Gilchrist S, Schooley MW, et al. Appraising the evidence for public health policy components using the quality and impact of component evidence assessment. Global Heart 2015;10(1):3–11; doi: 10.1016/j.gheart.2014.12.013. 25754561 PMC7333283

[12] Barbero C, Gilchrist S, Shantharam S, et al. Doing more with more: How “Early” evidence can inform public policies. Public Adm Rev 2017;77(5):646–649; doi: 10.1111/puar.12831. 32684642 PMC7367649

[13] Seidler A, Nußbaumer-Streit B, Apfelbacher C, et al. Rapid reviews in the time of COVID-19—Experiences of the competence network public health COVID-19 and proposal for a standardized procedure. Gesundheitswesen 2021;83(3):173–179; doi: 10.1055/a-1380-0926. 33634462 PMC8043586

[14] Chandra A, Martin L, Acosta J, et al. Transforming Public Health Data Systems, Who and What Next? Opportunities for a New Kind of Modernization. Robert Wood Johnson Foundation, National Commission to Transform Public Health Data Systems: Princeton, NJ; 2021.

[15] Association of Schools and Programs of Public Health. ASPPH Framing the Future: Education for Public Health 2030. Washington, DC; undated. Available from: https://www.aspph.org/teach-research/framing-the-future [Last accessed: April, 2022].

[16] Hanlon P, Carlisle S, Hannah M, et al. Making the case for a ‘fifth wave’ in public health. Public Health 2011;125(1):30–36; doi: 10.1016/j.puhe.2010.09.004. 21256366

[17] Baba C. University of Glasgow Institute of Health and Wellbeing Early Career Research's Blog. A ‘Fifth Wave’ in Public Health: Where Do We Start? Glasgow, Scotland; 2014. Available from: http://ihawkes.academicblogs.co.uk/2014/06/18/a-fifth-wave-in-public-health-where-do-we-start/ [Last accessed: April, 2022].

